# Sweet's Syndrome: A Case Report of a Rare Extraintestinal Manifestation of Ulcerative Colitis

**DOI:** 10.7759/cureus.22980

**Published:** 2022-03-08

**Authors:** Maria Inês Santos, Ana Mestre, Natália Teixeira, Cátia Correia, Marisa Brochado

**Affiliations:** 1 Internal Medicine, Hospital Distrital de Santarém, Santarém, PRT

**Keywords:** systemic corticosteroids, episcleritis, ulcerative colitis, inflammatory bowel disease, sweet’s syndrome

## Abstract

Sweet’s syndrome is a neutrophilic dermatosis of unknown etiology and a rare extraintestinal manifestation of ulcerative colitis.

Classically, it is more common in women with active inflammatory bowel disease (IBD). This syndrome typically presents in patients with acute-onset painful tender erythematous skin lesions and is usually accompanied by fever, arthralgia, and elevated inflammatory markers. Histological examination is characterized by diffuse dense dermal neutrophilic infiltrate with leukocytoclasia, without vasculitis.

The treatment goals are to reduce morbidity and complications, and the most effective therapy is systemic corticosteroids. Early recognition of this syndrome is essential to improve our diagnostic and therapeutic abilities.

We report a case of a 59-year-old female with ulcerative colitis, which presented with manifestations of Sweet’s syndrome.

## Introduction

Sweet’s syndrome (SS), also known as acute febrile neutrophilic dermatosis, is a rare skin condition characterized by the sudden appearance of a painful erythematous papular-nodular skin rash, commonly accompanied by fever and elevated inflammatory markers. Skin lesions are histologically characterized by neutrophilic infiltrate of the dermis without leukocytoclastic vasculitis [1].

The association between inflammatory bowel diseases (IBD) and SS was first described in 1988 by Kemmett et al. [2], and it is considered a rare extraintestinal manifestation of IBD. SS is less common in ulcerative colitis than in Crohn’s disease, and most cases associated with IBD occur in women with active disease [3].

The authors report a case of Sweet’s syndrome associated with a severe flare of ulcerative colitis.

## Case presentation

**Figure 1:**
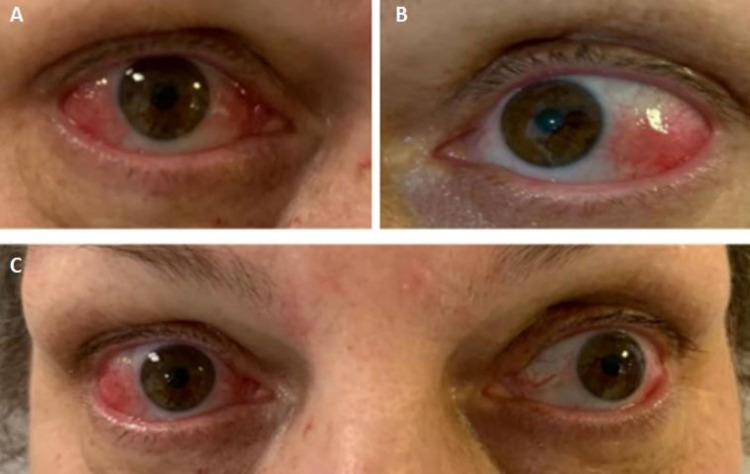
Ocular manifestation of Sweet’s syndrome Exuberant bilateral episcleritis (before treatment) A - right eye; B - left eye; C - both eyes

A 59-year-old female with a diagnosis of ulcerative colitis (UC) for less than a year, presented to the emergency department (ED) with a history of malaise, low-grade fever, painful (but non-pruritic) papulonodular skin lesions on her hands and thighs, and bilateral episcleritis (Figure 1) for the past five days. The patient also described intermittent abdominal pain and semi-fluid stools with mucus (stool frequency > 6 per day) two weeks before admission. She described no other constitutional symptoms and denied any recent travels, contact with pets, sick contacts, ingestion of unusual foods, or new medications. Since the diagnosis of UC, the patient was in remission on treatment with oral mesalazine 3 g/daily.

**Figure 2:**
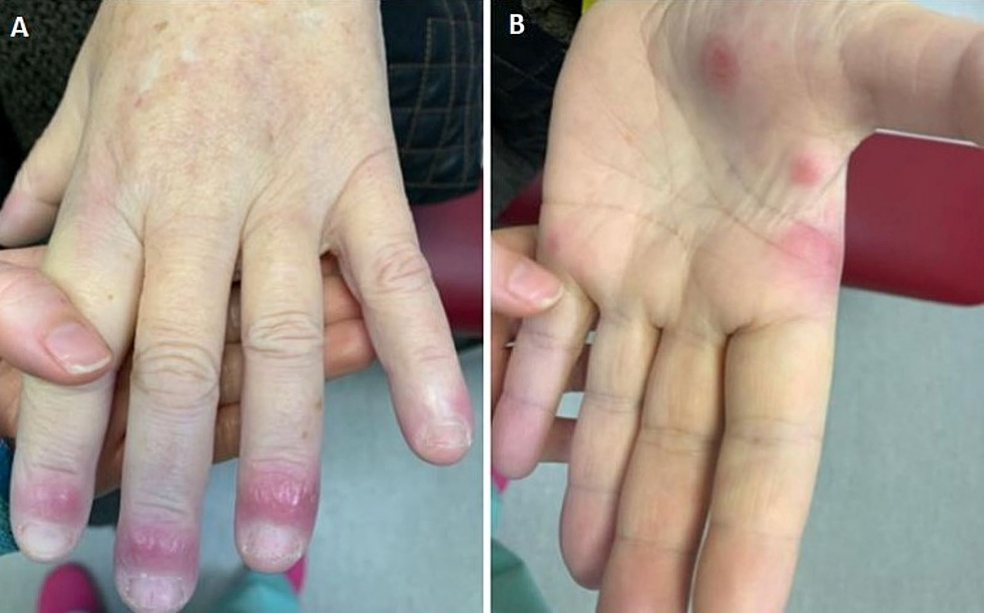
Cutaneous manifestation of Sweet’s syndrome Nodular, painful, and erythematous plaque lesions in her fingers (A) and palm (B)

**Figure 3:**
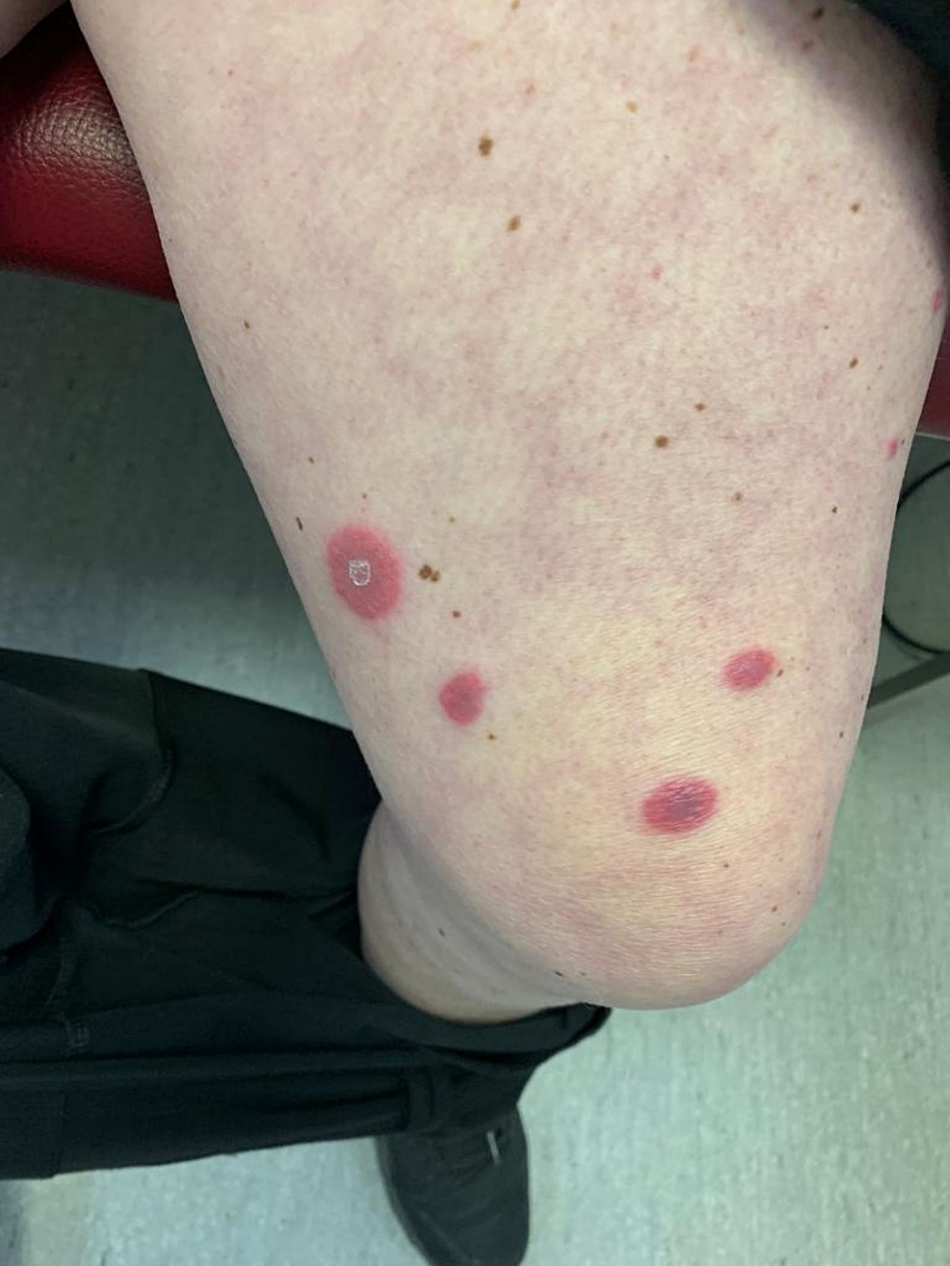
Cutaneous manifestation of Sweet’s syndrome Nodular, painful, infiltrative, and erythematous plaque lesions on the thigh (before treatment)

On admission, her physical examination was remarkable for fever (tympanic temperature of 38.2ºC), blood pressure 121/62 mmHg, heart rate 110/min, bilateral episcleritis, and painful violaceous erythematous skin papules and plaques mainly distributed on her hands (Figure 2) and anterior region of the thighs (Figure 3). On abdominal examination, mild tenderness to palpation was noted. There was no lymphadenopathy or signs of arthritis.

Laboratory evaluation revealed microcytic anemia (haemoglobin 9.3 g/L, mean cell volume 79 fL), leukocytosis (12700/ L), erythrocyte sedimentation rate of 72 mm/1st hour, and elevated C reactive protein of 18.16 mg/dL. Liver and renal functions, ionogram, and urinalysis were normal. Blood cultures were negative. Stool cultures and Clostridium difficile toxin were negative. The patient was negative for HIV 1 and 2, varicella-zoster virus, cytomegalovirus (IgM), Epstein-Barr virus (IgM), and hepatitis B and hepatitis C virus. Interferon-Gamma Release Assay (IGRA) was negative. C3, C4, rheumatoid factor, antinuclear antibody, and antineutrophil cytoplasmic antibodies tests were negative.

A computerized tomography (CT) scan was carried out, showing parietal concentric thickening of the rectal ampulla and approximately the entire sigmoid colon, with slight densification of adjacent fat and reduced colonic caliber, suggestive of inflammatory bowel disease.

**Figure 4:**
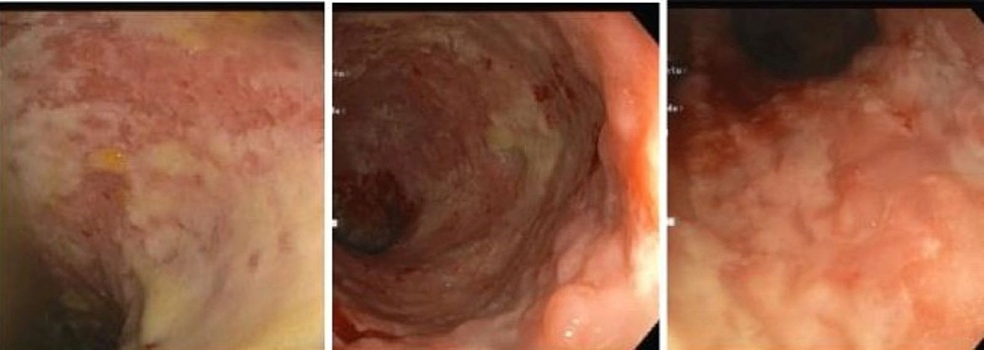
Colonoscopy Mucosa congestion, ulceration and mucopurulent adherent content due to ulcerative colitis flare

Colonoscopy was performed, showing congested and diffusely ulcerated colonic mucosa, with mucopurulent adherent content, revealing a severe flare of ulcerative colitis (Figure 4).

Ophthalmic examination showed bilateral episcleritis without complications. A skin biopsy of one of the lesions was performed and revealed an interstitial neutrophilic infiltrate in the dermis without vasculitis. All these findings were consistent with Sweet’s syndrome (SS) diagnosis.

**Figure 5:**
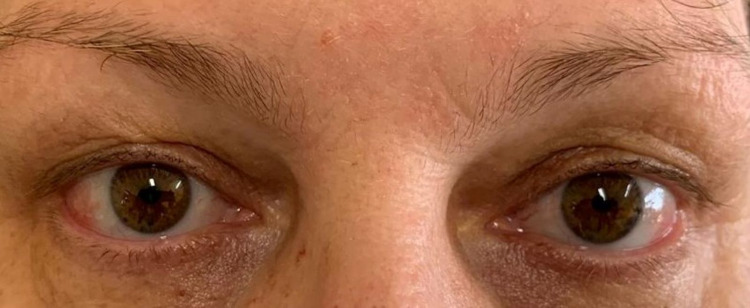
Evolution of episcleritis after five days of treatment

**Figure 6:**
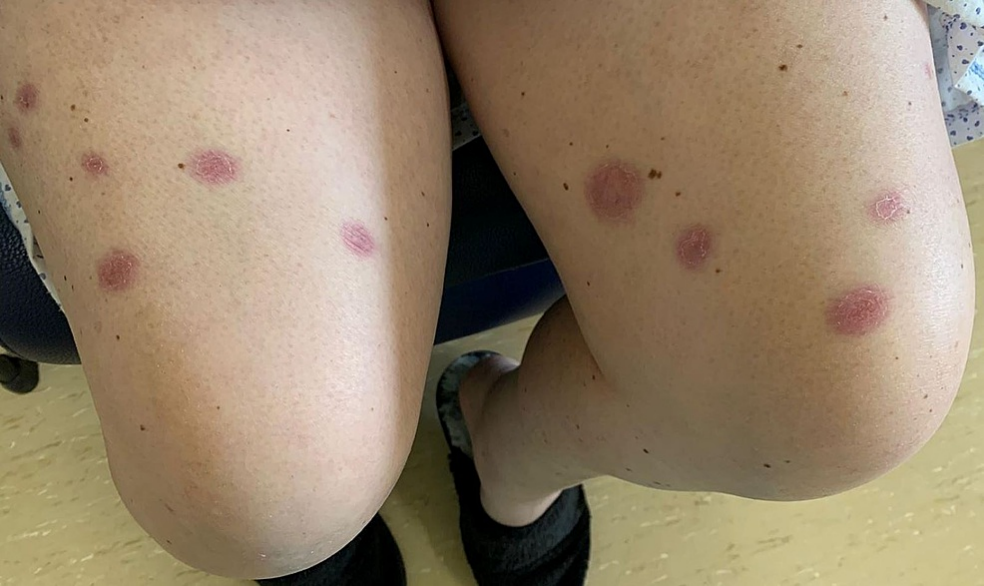
Cutaneous lesions of the thigh five days after treatment

Treatment with systemic corticosteroids was initiated with methylprednisolone 0.8 mg/kg/day, and improvement of the skin lesions and episcleritis was promptly observed (Figures 5-6). Stool frequency and consistency also improved over the course of treatment with corticosteroids. On the sixth day of admission, infliximab was initiated to remission UC, and methylprednisolone was switched to oral prednisolone. The patient was discharged on a prednisolone tapering regimen and infliximab on the tenth day.

After one year of regular follow-up examinations, no signs of recurrence of SS or flares of ulcerative colitis were noticed.

## Discussion

Sweet's syndrome was first described in 1964 by the British dermatologist Robert Douglas Sweet [4]. This condition is a neutrophilic dermatosis characterized by abrupt onset of painful erythematous skin nodules and papules accompanied by fever and leukocytosis with neutrophilia, with histopathological evidence of neutrophilic infiltrate of the dermis without vasculitis [4-5].

SS is more prevalent in women between 30 and 60 years old [2], and it is associated with various medical conditions such as malignancies (usually hematological), infections, pregnancy, inflammatory bowel diseases (IBD), and drugs. Thus, this disorder is classified into three clinical types: classical or idiopathic, malignancy-associated, and drug-induced Sweet's syndrome [6].

The underlying pathogenic mechanisms of Sweet's syndrome are multifactorial and remain poorly understood. It was proposed that certain diseases and drugs may induce a hypersensitivity reaction mediated by cytokines, increasing neutrophil activation, and resulting in fever and neutrophil infiltration of the dermis [6-8].

The main clinical manifestations of Sweet's syndrome are fever and skin lesions. Fever is the most frequent symptom; however, the cutaneous manifestations of the disease may precede it by several days to weeks [2]. Typical skin lesions commonly consist of painful, tender red or violaceous papules, nodules or plaques, asymmetrically distributed, affecting the face, neck, and upper extremities [6]. Besides these symptoms, this syndrome can also present with rare extracutaneous manifestations, such as arthralgias and arthritis, ocular inflammation (conjunctivitis and episcleritis), glomerulonephritis, and pleural effusion, particularly when associated with malignancy [3,9].

Histologically, the skin lesions are described as dense and diffuse mature neutrophils infiltrate in the reticular dermis, and it is differentiated from other types of dermatosis when dense neutrophilic infiltrate is seen without leukocytoclastic vasculitis [10].

In 1986, Su and Liu [11] proposed the first diagnostic criteria for classical SS, which were revised and modified by von den Driesch [12] in 1994 (Table 1). Diagnosis is confirmed when both major criteria and two of the four minor criteria are present.

**Table 1:** Diagnostic criteria for classic Sweet’s syndrome ESR - erythrocyte sedimentation rate; CRP - C-reactive protein

Major criteria:
1. Sudden onset of painful erythematous plaques or nodules.
2. Histologic evidence of dense neutrophilic infiltration in the dermis with no evidence of primary leukocytoclastic vasculitis.
Minor criteria:
1. Fever > 38°C
2. Excellent response to systemic corticosteroids or potassium iodide.
3. Abnormal laboratory values (3 of 4): ESR > 20 mm/h; high positive CRP; leukocytosis > 8000; with neutrophilia > 70%.
4. preceded by an upper respiratory infection or associated with hematologic or visceral malignancy, inflammatory disease or pregnancy.

Sweet’s syndrome is often associated with inflammatory bowel diseases, such as ulcerative colitis and Crohn’s disease, and is considered a rare extraintestinal manifestation of IBD. SS is more commonly observed in Crohn’s disease than in UC, with a higher prevalence in patients with colonic involvement and other extraintestinal manifestations [13-14].

The temporal relationship between SS and IBD activity is not well-defined, but it is most frequently associated with active IBD (67-80%). In one-third of the cases, Sweet’s syndrome may precede the symptoms or be present at the time of IBD diagnosis [14-15].

Untreated Sweet's syndrome lesions typically remain for weeks to months but eventually resolve in classical Sweet's syndrome patients within six to eight weeks [16].

There are no guidelines for managing Sweet's syndrome; however, systemic corticosteroids are the first-line treatment. Clinical manifestations usually respond promptly to this therapy, and symptoms start to resolve within 48 to 72 hours after initiation of corticosteroids [3]. The most described therapeutic regimen is prednisone at an initial dose of 0.5-1.5mg/kg/day, with a gradual tapering off over four to six weeks [3,6,17]. However, recurrence occurs in one-third of patients, and relapses are frequent if corticosteroids are tapered too rapidly; therefore, some patients need to maintain treatment for at least two to three months. Complete resolution of SS depends on the treatment of the underlying condition.

For extensive, severe, and resistant disease, as seen in our patient, in addition to the treatment of the underlying disease, intravenous corticosteroids with methylprednisolone were initiated with rapid improvement.

In patients who cannot be treated with corticosteroids, colchicine, and potassium iodide are also first-line agents [16]. Secondary therapies include indomethacin, cyclosporine, azathioprine, and dapsone [3,18]. There have been case reports of patients with IBD and refractory SS responding to monoclonal anti-TNF-α antibodies [19-20]. Then biologic therapy, such as infliximab, can represent an excellent therapeutic option for controlling severe cases of SS and IBD, especially if both are active simultaneously.

This case report highlights the importance of recognizing the association between Sweet's syndrome and inflammatory bowel disease.

## Conclusions

Sweet’s syndrome is a rare disorder associated with ulcerative colitis. Skin biopsy is essential for diagnosis, and systemic corticosteroid therapy is the gold standard treatment.

Sweet's syndrome should be considered an extraintestinal manifestation of UC; therefore, recognizing this association enables early diagnosis and appropriate treatment, which are essential to the successful management of this disease.
